# The Positive Role of Tai Chi in Responding to the COVID-19 Pandemic

**DOI:** 10.3390/ijerph18147479

**Published:** 2021-07-13

**Authors:** Suodi Xu, Julien S. Baker, Feng Ren

**Affiliations:** 1 Department of Physical Education, Ningbo University of Finance and Economics, Ningbo 315175, China; xusuodi@nbufe.edu.cn; 2 Centre for Health and Exercise Science Research, Department of Sport, Physical Education and Health, Hong Kong Baptist University, Hong Kong 999077, China; jsbaker@hkbu.edu.hk; 3 Faculty of Sports Science, Ningbo University, Ningbo 315211, China

**Keywords:** COVID-19, pandemic, Tai Chi, exercise intervention

## Abstract

The ongoing coronavirus 2019 (COVID-19) pandemic has posed a significant threat to both people’s physical and mental health. Physical inactivity, sedentary behavior, and negative emotions among the general population have been significantly increased because of COVID-19 home confinement. These are major risk factors associated with higher incidences of morbidity and mortality. Therefore, effective exercise management should be proposed as a prevention strategy to improve both physical and mental health while diminishing the effects of COVID-19. Tai Chi as a low-to-moderate aerobic exercise combines physical and mental training and plays a positive impact on human health. Here we aim to outline the effects of Tai Chi on the immune system, inflammatory responses, pulmonary function, and emotional control. The benefits of Tai Chi practice for individuals coping with COVID-19 are stated here which include immune system promotion, inflammation response reduction, rehabilitation in respiratory diseases, and emotional improvement. This statement has been supported by available clinical, physiological, and biological research. As a result, we hope to introduce Tai Chi as an effective exercise intervention for people coping with COVID-19 and as a beneficial exercise for maintaining an active lifestyle during a pandemic.

## 1. Introduction

Due to the consistently growing number of confirmed cases during the ongoing pandemic, the World Health Organization (WHO) appealed to all member states to implement effective measurements to curb human-to-human transmission. In response, the global public health departments of governments and health authorities have imposed extensive measures trying to mitigate and counteract the spread of COVID-19. These measures have included nationwide lockdowns, home confinement, social distancing, and self-isolation [1]. An inevitable consequence of these regulations was to restrict social activities in relation to access to public areas, gyms, parks, sports grounds, outdoor playing areas and schools. In addition, non-essential services were also closed to slow down the spread of the disease [2]. However, these quarantine strategies have negatively affected the health of the general population and have resulted in a reduction of physical activity (PA) and contributed to a sedentary lifestyle. Indeed, current reports show that people of various ages have faced difficulties maintaining at least similar levels of PA during home confinement [3,4,5,6].

In a recent international cross-disciplinary online survey about the changes in PA levels related to “before” and “during” confinement conditions, responders from Asia, Africa, Europe, and others indicated that the quantity of PA significantly reduced during home confinement. Specifically, the time performing vigorous intensity PA decreased by 33.1% minutes/day, moderate intensity decreased by 33.4% minutes/day, walking decreased by 34% minutes/day and combined activity decreased by 33.5% minutes/day [7]. As a result, daily sedentary time increased from 5 to 8 h per day due to reduced PA [7]. Importantly, both PA deficiency and sedentary behavior are associated with poor disease-related outcomes, such as an increase in the risk of the metabolic syndrome, type 2 diabetes, cardio-respiratory diseases, and some cancers [8,9,10,11,12].

Additionally, non-engagement in PA (failure to meet WHO PA recommendations) has been considered as an important risk factor for depressive symptoms developed during the confinement period. As a result, anxiety, frustration, depression, sleep disorders, and domestic violence have become quite common during home confinement with helpline numbers being overloaded [13,14]. In the international cross-disciplinary online survey conducted by Ammar et al., it was found that the COVID-19 home confinement period induced an adverse effect on mental health with a greater proportion of respondents suffering from psychosocial stress and emotional disorders. Previously, during the outbreak of “severe acute respiratory syndrome” (SARS), emotional and psychological issues in quarantined individuals have been a concern. It has been revealed that the individuals who experienced self-isolation suffered from more anxiety and tension [15,16]. Generally, lack of PA and mental health disorders are among the major risk factors associated with the incidence of morbidity and mortality [17,18]. As a result, effective home-based exercise programs should be proposed to improve people’s physiological health, mental health, and quality of life during home confinement. Here, we would recommend Tai Chi as a potential exercise intervention to improve people’s healthy lifestyle during a pandemic. Tai Chi is one of the Chinese martial arts consisting of light-to-moderate aerobic exercise combining both physical and mental training [19]. In fact, Tai Chi has been suggested as a feasible program for promoting active lifestyles during pandemics previously [20]. Here we will discuss the benefits of Tai Chi in coping with the ongoing pandemic in terms of immune system, inflammatory responses, lung function, and mental health.

## 2. The Benefits of Tai Chi on Human Health

**Figure 1:**
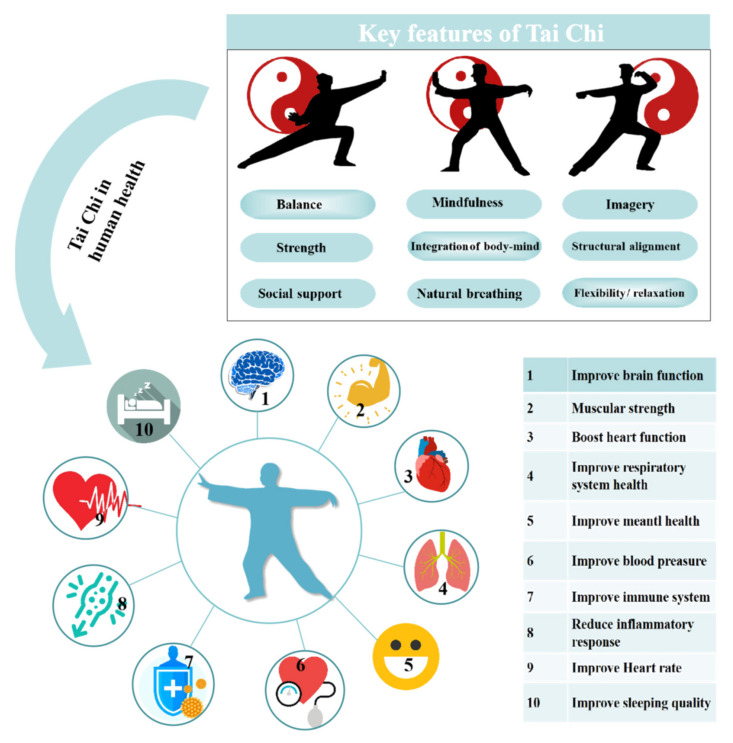
Key features of Tai Chi and it is influence on human health.

The mindfulness and flexibility movements of Tai Chi which integrates multiple features has become a popular exercise around the world—the key features of Tai Chi are presented in Figure 1 [19]. Researchers indicate the significant value of Tai Chi in promoting physical health and benefits the practitioners with various positive health outcomes including muscular strength, aerobic capacity, balance and motor control, prevention of falls, mental health, sleep disorders, fatigue, body mass index, blood pressure, heart rate, etc. (Figure 1) [21]. Tai Chi is already prescribed in therapeutic programs for patients with neurological diseases (Parkinson’s disease, traumatic brain injury, multiple sclerosis), rheumatological disease (rheumatoid arthritis, ankylosing, spondylitis, fibromyalgia), orthopedic disease (osteoarthritis, osteoporosis, low-back pain, musculoskeletal disorder), cardiovascular disease (acute myocardial infarction, coronary artery bypass grafting, congestive heart failure), certain cancers, as well as a pulmonary disease [22].

## 3. Effect of Tai Chi Practice on Immune Function and Inflammation

Immune system function and inflammatory biomarker responses can be modulated by Tai Chi practice. Regarding the immune system, Tai Chi can increase the levels of immune cells in both innate immune systems and adaptive immune systems. Liu et al. observed a Tai Chi training group with 30 middle-aged participants who practiced Tai Chi for 6 months, four times a week, for 60 min. They found a significant increase in the percentage of natural killer (NK) and NKT cells compared with a control group. There was also T cell improvement, resulting in a higher level of Th1 immune responses and therefore potentially increased anti-viral function in humans [23]. In addition, a meta-analysis review of 19 studies based on the effects of Tai Chi and qigong (TQ) training on immune responses found similar results, concluding that TQ presented a significant small effect in enhancing the immune cell levels as well as inflammatory processes [24]. The positive effects of Tai Chi on immune response have also been confirmed in antibody levels following vaccination. Yang et al. suggested that the traditional TQ training could increase the antibody response [25].

Regarding the impacts of Tai Chi interventions on inflammation response, the levels of C-reactive protein reduced following Tai Chi training, which is a common diagnostic maker used to estimate systemic inflammation [26]. Additionally, the level of cell-mediated inflammatory cytokines (IL6, TNF-α) were generally reduced in a Tai Chi intervention group compared to the control group [27,28,29]. Indeed, cellular interactions in the inflammatory mediators, innate immune system, and adaptive immune system induce various aspects of acute and chronic inflammation in many organ diseases [30].

Clinically, COVID-19 is characterized by an overexuberant inflammatory response— it is a necessary response of the immune system to infection. The system then activates a coagulation reaction resulting in excessive production of proinflammatory cytokines (IL-6, IL-1b, and TNF-α) causing multiorgan injury, leading to acute respiratory distress syndrome [31,32]. The levels of inflammatory cytokines (IL6, TNF-α) can be reduced during Tai Chi practice which has been demonstrated in many randomized controlled trials. Based on these findings, Tai Chi can be recommended as an appropriate exercise intervention to reduce inflammation response, improve the immune system, and resist viral infections. In a recent clinical case report outlining the case of a patient diagnosed with mild symptoms of COVID-19, the doctor advised the patient to practice Tai Chi as a rehabilitation therapy when the patient was in recovery [33]. However, in future studies, a large randomized controlled trial is needed to confirm the prevention and improvement effects of Tai Chi on COVID-19 in humans.

## 4. Application of Tai Chi to Rehabilitation in Pulmonary Function

Tai Chi training as a pulmonary rehabilitation program has shown the positive effects on patients with chronic obstructive pulmonary disease (COPD) [34,35,36,37,38,39]. COPD is a disease characterized by restricted airflow due to abnormal airways and/or alveolar, accompanied by chronic symptoms such as dyspnea, cough, and sputum production [34]. Currently, Tai Chi practice has been considered as an effective exercise intervention to improve lung function in patients with COPD around the world [35,36]. Breathing techniques are very important during Tai Chi exercise which coordinates balance and strength training. The style of mind-body breathing as a component of Tai Chi provides lung function improvement for the COPD population. Lung capacity could be enhanced by this kind of breathing strategy which combines slow and deep breathing to encourage a complete exhalation [37,38,39]. Additionally, the strength of the respiratory muscles could also be increased using mind-body breathing [37].

Additionally, a Cochrane review has shown a positive impact of Tai Chi training on pulmonary function in people with COPD compared to conventional treatment and outlined four collected studies with 570 subjects presenting an improvement of FFV1 (forced expiratory flow volume in one second) and FVC (forced vital capacity) in post-program data [40]. Researchers have investigated the effects of long-term home-based Liuzijue exercise on COPD patients. Liuzijue is a low-moderate exercise similar to Tai Chi which has more focus on abdominal breathing and pursed lip breathing. Following 6 months of Liuzijue intervention, pulmonary function (FEV1/pred, FEV1/FVC) significantly improved in training individuals when compared with a control group [41]. It was suggested that a combination of Liuzijue exercise and clinical guidance can be used as a feasible and effective method to improve lung function, exercise capacity, as well as the quality of life in elderly patients with COPD.

## 5. Effects of Tai Chi Intervention on Negative Emotions (Anxiety and Depression)

Tai Chi emphasizes body-mental training that coordinates body-based exercise and mind-based practice. Body-based exercise focuses on body control with a state of musculoskeletal relaxation, whereas mind-based practice emphasizes the mental control of concentration in a mindfulness way [42,43,44]. Most studies have shown a preventive effect of Tai Chi training on reducing negative emotions, such as depression, anxiety, and sadness in many clinical studies [45,46,47]. Kimberly and colleagues conducted a 10-week pilot study in which they recruited 24 patients with a mean age of 52 years with varying levels of anxiety, as measured by the Hamilton Anxiety psychiatric rating scale. The authors were surprised to find that participants’ emotion improved dramatically, and anxiety reduced by 84% after attending a 10-week program of Tai Chi classes [48]. This strongly indicates that Tai Chi could be used as an effective method for reducing anxiety. Additionally, the influence of Tai Chi on negative mental conditions in non-clinical populations by using meta-analysis has been investigated by Zhang et al. [49] Fourteen experimental studies were evaluated, and the results showed that the negative emotions in both young and old adults could be significantly improved by practicing Tai Chi. The authors suggested that Tai Chi can be used as an effective complementary non-pharmacological resource in coping with anxiety and depression. Researchers have also explored the motivations of Tai Chi training among 35 survivors who were infected with SARS-CoV-1 during the 2003 outbreak in Hong Kong; the study showed that almost all of the subjects obtained relief from their emotional suffering of psychological sequelae through Tai Chi training [50].

The positive effect of Tai Chi on improving negative emotions could be explained from several aspects. Firstly, the mindfulness function of Tai Chi, with an emphasis on attention control and self-awareness to improve meditative levels during Tai Chi training, may contribute to depression and anxiety reduction [51,52,53]. Secondly, the abdominal breathing function associated with Tai Chi practice may have an impact on heart rate variability. This can be improved by increasing breathing amplitude in combination with abdominal breathing patterns, which are used as a psychophysiological marker of the brain’s capacity to regulate emotional responses [54,55,56]. Thirdly, Tai Chi has a role in exercise-induced brain-derived neurotrophic factor. Tai Chi, as an aerobic exercise program of light-to-moderate intensity can increase the production of insulin-like growth factor and brain-derived neurotrophic factor which are neurobiological markers related to emotional regulation [57,58,59,60]. In general, the effects of Tai Chi on emotional improvement has been confirmed by some researchers, and it could be an effective method to improve mental health in both young and older adults during home confinement.

## 6. Conclusions

The available clinical and biological research supports that Tai Chi can be used as an effective exercise intervention to improve both physical and psychological health. Some of the benefits of Tai Chi practice for individuals coping with COVID-19 are outlined here. Benefits include immune system promotion, inflammation response reduction, rehabilitation in pulmonary function, and emotional improvement. These positive effects of Tai Chi on human health can be recommended as alternatives to counteract the negative effect of physical inactivity, sedentary behavior, and mental disorders on the general population during the confinement period. Furthermore, Tai Chi may be useful in achieving prevention, to treat the condition, and provide rehabilitation following COVID-19 infection. Additionally, Tai Chi is easy to practice and is safe to perform at home, in isolation, or in groups. It is suitable for the elderly, individuals with emotional disorders, and chronically ill populations. Future research is required to confirm the effectiveness of Tai Chi during the current pandemic and to provide more valid and reliable data on this topic.
